# Health-Related Quality of Life (HRQoL) Outcomes Following Injury in Childhood and Adolescence Using EuroQol (EQ-5D) Responses with Pooled Longitudinal Data

**DOI:** 10.3390/ijerph181910156

**Published:** 2021-09-27

**Authors:** Joanna F. Dipnall, Frederick P. Rivara, Ronan A. Lyons, Shanthi Ameratunga, Mariana Brussoni, Fiona E. Lecky, Clare Bradley, Ben Beck, Jane Lyons, Amy Schneeberg, James E. Harrison, Belinda J. Gabbe

**Affiliations:** 1 School of Public Health and Preventive Medicine, Monash University, Melbourne 3004, Australia; r.a.lyons@swansea.ac.uk (R.A.L.); s.ameratunga@auckland.ac.nz (S.A.); ben.beck@monash.edu (B.B.); belinda.gabbe@monash.edu (B.J.G.); 2 Institute for Mental and Physical Health and Clinical Translation, School of Medicine, Deakin University, Geelong 3220, Australia; 3 Departments of Pediatrics and Epidemiology, Harborview Injury Prevention and Research Center, University of Washington, Seattle, WA 98195, USA; fpr@u.washington.edu; 4 Health Data Research UK, Swansea University, Swansea SA2 8PP, UK; j.lyons@swansea.ac.uk; 5 National Centre for Population Health and Wellbeing Research, Swansea University, Swansea SA2 8PP, UK; 6 School of Population Health, University of Auckland, Auckland 1023, New Zealand; 7 Kidz First Hospital and Population Health Directorate, Counties Manukau District Health Board, Auckland 2025, New Zealand; 8 Department of Pediatrics, School of Population and Public Health, University of British Columbia, Vancouver, BC V6H 3V4, Canada; mbrussoni@bcchr.ubc.ca; 9 British Columbia Injury Research and Prevention Unit, British Columbia Children’s Hospital Research Institute, Vancouver, BC V6H 3V4, Canada; amyschneeberg@gmail.com; 10 Human Early Learning Partnership, School of Population and Public Health, University of British Columbia, Vancouver, BC V6T 1Z3, Canada; 11 Centre for Urgent and Emergency Care Research, School of Health and Related Research, University of Sheffield, Sheffield S10 2TN, UK; f.e.lecky@sheffield.ac.uk; 12 Emergency Department, Salford Royal Hospital, Salford M6 8HD, UK; 13 South Australian Health and Medical Research Institute, Adelaide 5001, Australia; Clare.Bradley@sahmri.com; 14 College of Medicine and Public Health, Flinders University, Bedford Park 5042, Australia; james.harrison@flinders.edu.au; 15 School of Population and Public Health, University of British Columbia, Vancouver, BC V6T 1Z3, Canada

**Keywords:** health-related quality of life, health outcomes, disability, trauma, injury, EQ-5D, children, pediatric, adolescents

## Abstract

Background: Injury is a leading contributor to the global disease burden in children, affecting their health-related quality of life (HRQoL)—yet valid estimates of burden are absent. Methods: This study pooled longitudinal data from five cohort studies of pediatric injury survivors (5–17 years) at baseline, 1-, 4-, 6-, 12-, and 24- months (*n* = 2334). HRQoL post-injury was measured using the 3-level EQ-5D utility score (EQ-5D) and five health states (mobility, self-care, activity, pain, anxiety and depression (anxiety)). Results: Mean EQ-5D post-injury did not return to baseline level (0.95) by 24 months (0.88) and was lower for females over time (−0.04, 95%CI −0.05, −0.02). A decreased adjusted risk ratio over time (ARR) was observed for intentional injuries (pain: 0.85, 95%CI 0.73,0.98; anxiety: 0.62, 95%CI 0.49,0.78); spinal cord injuries (mobility: 0.61, 95%CI 0.45,0.83), self-care: 0.76, 95%CI 0.63,0.91, activity: 0.64, 95%CI 0.47,0.88); moderate/severe traumatic brain injury (activity: 0.83, 95%CI 0.71,0.96). ARRs were also low for certain fractures, with various health states affected. Conclusions: HRQoL outcomes over time for children and adolescents post-injury differed across key demographic and injury related attributes. HRQoL did not reach levels consistent with full health by 24 months with recovery plateauing from 6 to 24 months. Tailored interventions are required to respond to the varying post-injury recovery trajectories in this population.

## 1. Introduction

Injury is a leading contributor to the global disease burden in children, placing them at risk of long-term adverse impacts on their health-related quality of life (HRQoL) [1,2]. Various methods are used to estimate burden of injury in this group and the absence of valid estimates of the burden of nonfatal injury in children and adolescents has meant that there has been limited capability to accurately quantify the HRQoL.

Measuring HRQoL following injury is an important basis for exploring pathways to recovery. Generic and easily administered multi-attribute utility instruments (MAUIs) have been used for both recovery and health economic analyses. The EuroQol Group’s EQ-5D [3] provides an overall measure of HRQoL and five health states or domains, which is the preferred MAUI for estimating recovery over time [4], cost-utility analysis [5] and use across a variety of settings and applications [6].

The EQ-5D is a standardized measure of health status based on responses to questions about problems (none, some, extreme) in five domains (mobility, self-care, activity, pain or discomfort, and anxiety and depression). An overall utility value can be calculated from the responses. The adult version of the EQ-5D has been used to assess HRQoL after injury in adults [4] and has found problems persisting up to three years post-injury. The EQ-5D has been found to be an appropriate instrument for collecting health related quality of life data among injured children [7] but there have been few studies in children and adolescents that address a diversity of injury types, primarily due to sample size limitations. Obtaining valid estimates of HRQoL overall and for different health states, and trajectories of recovery over time, has the potential to inform researchers and policy makers and guide interventions.

The Validating Injury Burden Estimates Study for children (VIBES-Junior) pooled longitudinal data across a number of time points from five cohort studies of pediatric injury survivors. The aim of this study was to use the EQ-5D to (1) characterize and establish the key predictors of overall HRQoL and health states following injury in childhood and adolescence, and (2) to predict HRQoL recovery trajectories for different health states over time post-injury.

## 2. Materials

### Patient Cohorts

The VIBES-Junior protocol is published elsewhere [8]. A summary of the five cohorts included in this pooled analysis is outlined below:

1. The Victorian State Trauma Registry (VSTR) is a population-based trauma registry that captures data about all major trauma patients in the state of Victoria in Australia [9];
2. The Victorian Orthopedic Trauma Outcomes Registry (VOTOR) is a clinical registry of orthopedic injuries, treatment, complications and outcomes based on admissions to four Australian centres [10].
3. The US Children’s Health After Injury (CHAI) study included children with mild, moderate and severe traumatic brain injury (TBI) or with upper extremity injuries who presented to a set of US hospitals [11].
4. The United Kingdom Burden of Injury (UKBOI) was a study of injured individuals with children recruited from emergency department (ED) presentations and hospital admissions in four UK centers [12].
5. The British Columbia Children’s Hospital Longitudinal Injury Outcomes (BCCH-LIO) study included children who attended the British Columbia Children’s Hospital in Canada for an injury [13].

The full rationale for study inclusion is provided in the published protocol. In summary, these injury specific longitudinal studies were included as they collected HRQoL outcomes at multiple time points after injury to give a comprehensive evaluation of the long-term impact of injury in childhood and adolescence and enable comparison across all injury types. These cohort studies were integrated using the data integration protocol in ten steps (DIPIT) [14] and each cohort is outlined in Table 1.

## 3. Methods

### 3.1. Measures

#### 3.1.1. Demographic and Injury Characteristics

Demographic and injury characteristics were collected at baseline and included sex (male, female); age group, split into three categories to align with the World Health Organization (WHO) classification [2] within the age band of our pooled cohort of 5 to 17 years (5–9 years, 10–14 years, 15–17 years); and a measure of socio-economic status (SES). Each VIBES-Junior cohort had a different measure of SES: VSTR and VOTOR contained quintiles of the Index of Relative Socio-economic Advantage and Disadvantage (IRSAD), which is an area-based measure released by the Australian Bureau of Statistics; CHAI contained quintiles created from principal components analysis using income and education variables from the CHAI data; UKBOI contained quintiles from Townsend Deprivation Score reversed so that higher quintiles reflect higher SES; and BCCH-LIO initially contained quintiles from Quintile of Annual Income Per Person Equivalent (QAIPPE), which is an area-based measure released by Statistics Canada. The measures of SES were collapsed from quintiles into tertiles (low, moderate, high).

Mechanism of injury was dichotomized to injuries sustained during transport (motor vehicle occupant, pedestrian, or on a motorcycle or bicycle) and non-transport injuries (falls, struck by/against an object or person, and other mechanisms). A binary measure of emergency department (ED) presentation and discharge = 0 versus hospital admission = 1 was created. Injury severity score was collapsed into tertiles (low, mid, high) where the cut points were based on each cohort. The ISS is considered the most widely used to assess trauma severity. [15,16] Intent of injury was grouped into three groups (intentional (including self-harm, maltreatment and interpersonal violence), unintentional, and intent not known).

Diagnoses and external cause codes were classified using the ICD 10th Revision (ICD-10). The CHAI data were mapped from the ICD 9th Revision (ICD-9) to the ICD-10. All diagnosis codes were mapped to the 2013 Global Burden of Disease (GBD) study injury health states as this report contained tables related to these classifications, enabling cross-walking from the ICD-10 codes to the GBD injury groups. Injury groups were collapsed into 17 binary variables indicating the presence or absence of that injury: N33, N34 spinal cord lesion, N19, N26 fracture of femur, N20 fracture of patella, tibia, fibula, or ankle, N28 moderate to severe TBI, N37, N17, N18 crush injury, fracture foot/hand bones, N43 internal hemorrhage in abdomen or pelvis, N27 minor TBI, N21 fracture of pelvis, N42 severe chest injury, N8, N9, N10 burns (including lower airways), N25 fracture of vertebral column, N35, N36 asphyxiation, non-fatal submersion, N40, N44 contusion, open wound, N14 other injuries of muscle & tendon and other dislocations, N15 fracture of clavicle, scapula, or humerus, N22 fracture of radius or ulna, and other. The other group included injuries such as amputation of one limb/toe, poisoning, injured nerves, environmental factors, injured nerves, dislocation of shoulder/hip/knee, fracture of ribs/sternum/skull/face bone, foreign body in ear/gastrointestinal or urogenital system, superficial injury, and injury to eyes. Comorbidities present at the time of injury was based on the 27 health conditions described by Mitchell et al. [17] and were collapse into two groups (no comorbidities or ≥1 comorbidities).

#### 3.1.2. EQ-5D

This study used the three-level EQ-5D scale (EQ-5D-3L) to represent the measures of overall HRQoL and the five health states of HRQoL (Supplementary Table S1). For consistency across the cohorts, parent item scores were used. Four of the five cohorts collected the EQ-5D outcomes at multiple time points after injury (VSTR, VOTOR, UKBOI, BCCH-LIO). The CHAI study collected the Pediatric Quality of Life Inventory (PedsQL) and so responses to these questions were mapped to the EQ-5D using the algorithm developed by Khan et al. [18] An overall EQ-5D utility score was created from the five health state questions and used country specific age- and gender- specific population weights or tariffs (https://euroqol.org/eq-5d-instruments/eq-5d-3l-about/valuation/ (accessed on 16 June 2021)). Values of the EQ-5D utility score range from 0 = a state as bad as being dead to 1 = full health, and negative EQ-5D utility score values represent health states regarded as worse than a state as bad as being dead.

The original three level descriptive system for each EQ-5D health state were: no problems, some problems, and extreme problems. Due to low frequency across some and extreme problems, a binary measure was created where 1 = no problems and 0 = problems. the *some problems* category included both *some problems* and *extreme problems* responses. As there was no map available of the individual EQ-5D-3L health state questions from the PedsQL, the CHAI cohort was excluded from this analysis.

#### 3.1.3. Time Points

Longitudinal analysis of the EQ-5D utility score examined five time points: 1 month; 3 to 4 months; 6 months; 12 months; and 24 months. This analysis included all pooled data from all five VIBES-Junior cohorts. The 3 to 4 months data include both the BCCH-LIO and CHAI cohorts and were used to identify the short-term overall HRQoL burden post-injury.

Reflecting the absence of the CHAI cohort, longitudinal analysis of the EQ-5D five health states included four time points: 1 month; 6 months; 12 months; and 24 months. Due to sample size issues with the distribution of the binary EQ-5D health state questions, the 4-month BCCH-LIO data were included in the 6-month time point.

### 3.2. Statistical Analysis

Means and standard deviations of the continuous variables and frequencies and percentages of the categorical measures were reported. Comparison with healthy population norms was not conducted as there were no EQ-5D-3L healthy population norms available for children and adolescents [19]. Participants were considered lost to follow-up if the EQ-5D utility score and/or EQ-5D health state binary items were missing across all time points post-injury or if the patient had died following hospital discharge.

Patterns of EQ-5D health state responses were quantified for each time point as described by Devlin et al. [20]. The distribution of the ten most common response patterns from 1 month was graphed and compared to the response patterns in months 6, 12 and 24. This was done because changes in the distribution of health profiles or states over time may provide insights into issues with certain EQ-5D health states. Health state density curves (HSDC) were created for EQ-5D health state patterns across each time point [21]. These plots compared the cumulative frequency of health states against the cumulative frequency of the sample at each time point. Values following a 45-degree line would indicate an even spread of health states across the sample. Observed values are well below this line, indicating that health states are unevenly distributed.

Mixed effect regression models were used to predict outcomes [22]. Missing data on the covariates included in the models was quantified and found to be acceptable at <5% [23,24]. Mixed effects regression has been shown to be flexible in handling missing data compared to using multiple imputation which has been found to potentially produce unstable results [25]. Time was treated as a discrete categorical variable, requiring no assumptions to be made about its mathematical function.

Demographic and injury-related risk factors identified in prior research were included in all models. The EQ-5D utility score was modelled using a mixed effects linear regression with random intercepts and slopes to adjust for the correlation between patients. Interactions between time and sex and time and age group were tested to establish if outcome trajectories differed according to the patient’s sex or age. Adjusted mean differences with 95% confidence intervals were produced for the mixed effects linear regression. The five separate binary EQ-5D health state items were modelled using a modified Poisson model with random intercepts to estimate relative risks (RR) for each binary outcome [26]. Adjusted RR with 95% confidence intervals were produced for the mixed effects modified Poisson models. Predicted margins across time with 95% confidence intervals were estimated and graphed for the EQ-5D utility score across sex and age group and overall across time for each of the five EQ-5D health states.

All analyses were performed using Stata version 16.0 (Stata Corp, College Station, TX). All models used robust standard errors to allow for potential heteroscedastic residuals. The 95% confidence intervals were evaluated for effect size and significance for all analyses.

### 3.3. Ethics

The project was approved by the Monash University Human Research Ethics Committee (project number 12311) and was conducted in compliance with the NHMRC National Statement on Ethical Conduct in Human Research (2007)—Updated 2018 [27] and the ICH Guideline for Good Clinical Practice E6(R2).

## 4. Results

### 4.1. Pooled Cohort Overview

A total of 2334 children and adolescents were included in the pooled analysis (Table 2). A higher proportion of cases who were lost to follow up were younger, from the VSTR cohort, had at least one comorbidity, had a higher ISS, or an intentional injury. The majority of the pooled cohort included in the analysis were male (73%) and 53% were aged between 15 and 17 years, with a mean age of 13.6 years (SD = 3.5). The majority of the included cases were from moderate to high SES (62%), had a non-transport related injury (63%), had a hospital admission (73%), and had no comorbidities recorded at the time of their injury (92%).

### 4.2. EQ-5D

**Figure 1:**
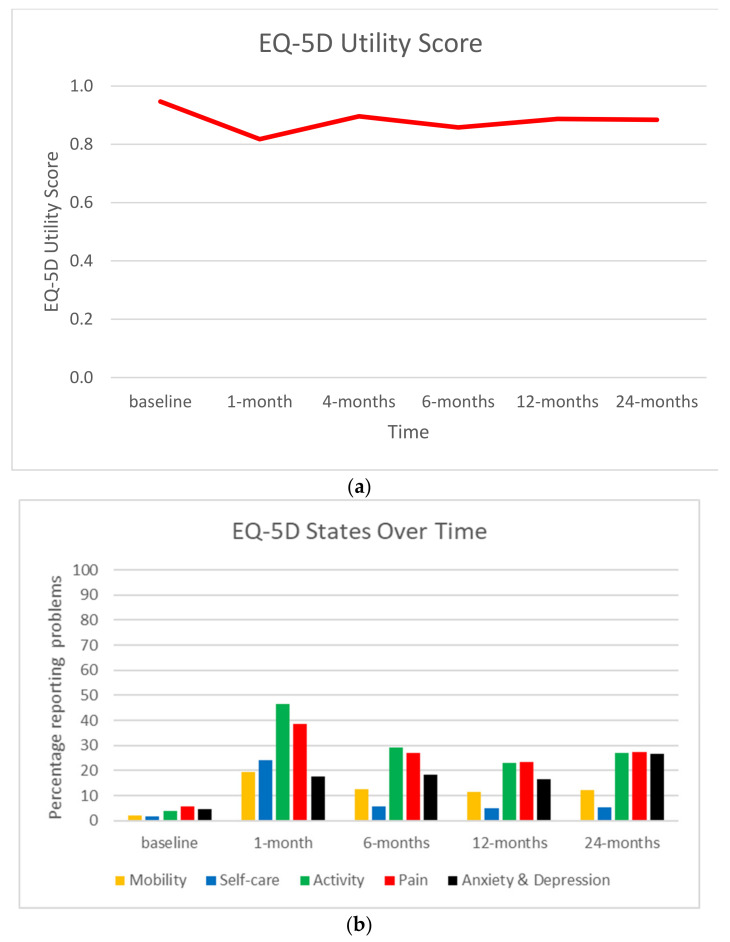
EQ-5D over time. (**a**) EQ-5D utility score. (**b**) Percentage of participants reporting problems (some or extreme).

The mean EQ-5D utility score was lowest at 1 month post injury, rising at 4 months and plateauing between 6 months and 24 months, never reaching the level of baseline (Supplementary Table S2, Figure 1a). At 1 month post-injury the percentage of problems were highest for mobility, self-care, activity, and pain, but not anxiety and depression (Supplementary Table S2, Figure 1b), which was highest at 24 months. The proportion of children having problems with activity, pain and anxiety and depression rose by ≥1% between 12 months and 24 months.

**Figure 2:**
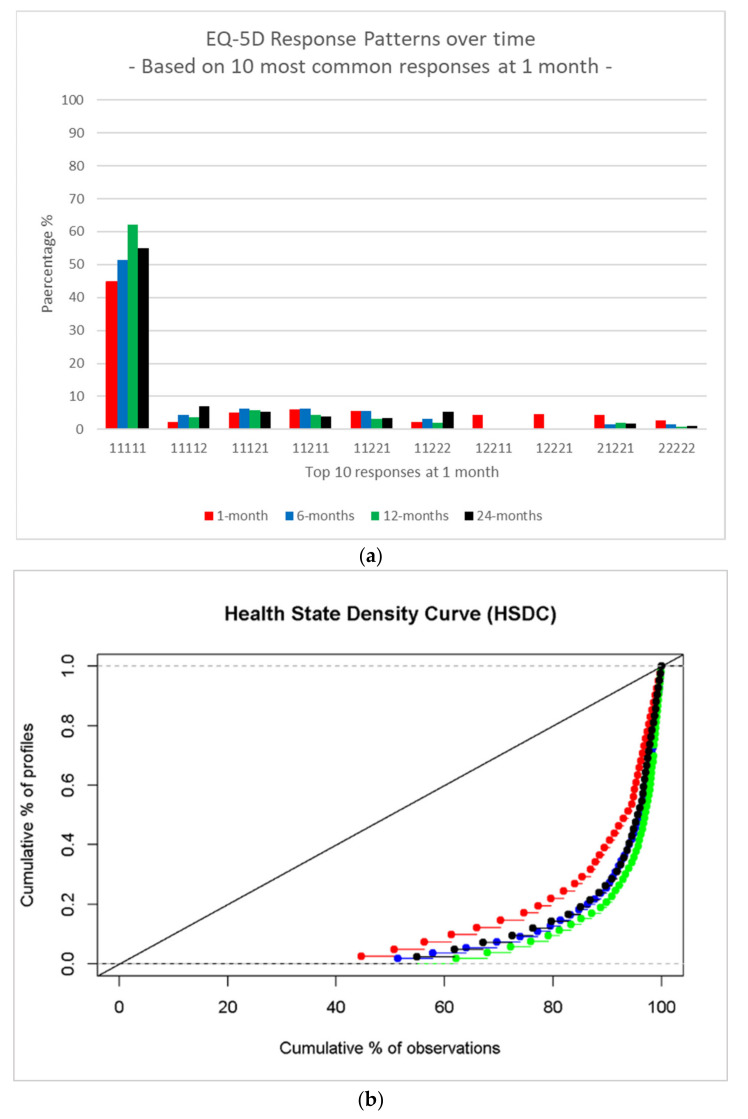
(**a**) EQ-5D health state response patterns over time based on the 10 most common responses in 1 month. 1 = no problems, 2 = problems. The pattern order for health states = (mobility self-care activity pain, anxiety, and depression). (**b**) Health state density curve (HSDC) across time: red = 1 month, blue = 6 months, green = 12 months, black = 24 months.

The proportion of children with a complete health state response pattern with no problems (i.e., 11111) was lowest at 1 month (45%) and highest at 12 months (62%) but declined at 24 months (55%) (Figure 2a). There was a rise at 24 months for the health states 11112 and 11222, which included problems with pain in both these health state patterns. The HSDC graph indicates the health states were more evenly spread at 1 month (red) and less evenly spread at 12 months (green) (Figure 2b).

### 4.3. EQ-5D Utility Score Model

**Figure 3:**
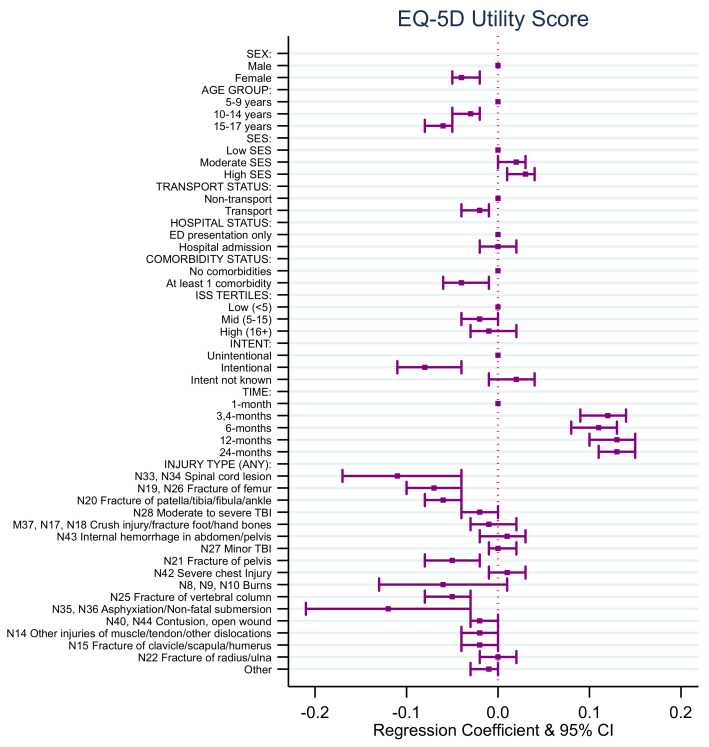
EQ-5D utility score regression coefficients.

Results from the EQ5-D utility score regression model indicated that, on average, females had a lower EQ-5D utility score post-injury over time compared to males (Supplementary Table S3, Figure 3). The average EQ-5D utility score post-injury over time for children aged 10–14 years of age and aged 15–17 years of age was lower than those aged 5–9 years of age. Children from moderate and high SES areas had a higher average EQ-5D utility score post-injury over time compared to those from low SES.

Children involved in a transport-related injury had a lower average EQ-5D utility score post-injury over time compared to children who were involved in a non-transport injury (Supplementary Table S3, Figure 3). Children involved in intentional injuries had a lower average EQ-5D utility score post-injury over time compared to children involved in unintentional injuries.

Children who had sustained a fracture of the femur, patella/tibia/fibula/ankle, pelvis, clavicle/scapula/humerus, and/or vertebral column had a lower average EQ-5D utility score post-injury over time compared to children who had not sustained these types of injuries (Supplementary Table S3, Figure 3). Children who had sustained a moderate to severe traumatic brain injury had a lower average EQ-5D utility score post-injury over time compared to children who had not sustained this type of injury. Children who had sustained a non-fatal submersion injury resulting in asphyxiation had a lower average EQ-5D utility score post-injury over time compared to children who had not sustained this type of injury.

Children who had sustained a spinal cord injury had a lower average EQ-5D utility score post-injury over time compared to children who had not sustained this type of injury (Supplementary Table S3, Figure 3). Children who sustained a contusion or open wound had a lower average EQ-5D utility score post-injury over time compared to children who had not sustained these types of injury. Children who had sustained other injuries of muscle and tendon and other dislocations had a lower average EQ-5D utility score post-injury over time compared to children who had not sustained this type of injury.

### 4.4. EQ-5D Health State Models

**Figure 4:**
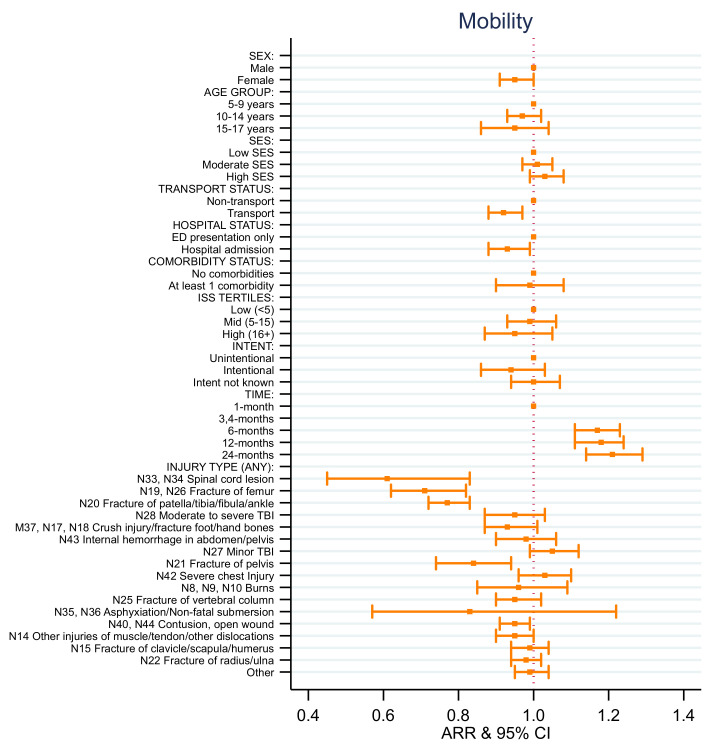
EQ-5D mobility adjusted relative risk (ARR).

**Figure 5:**
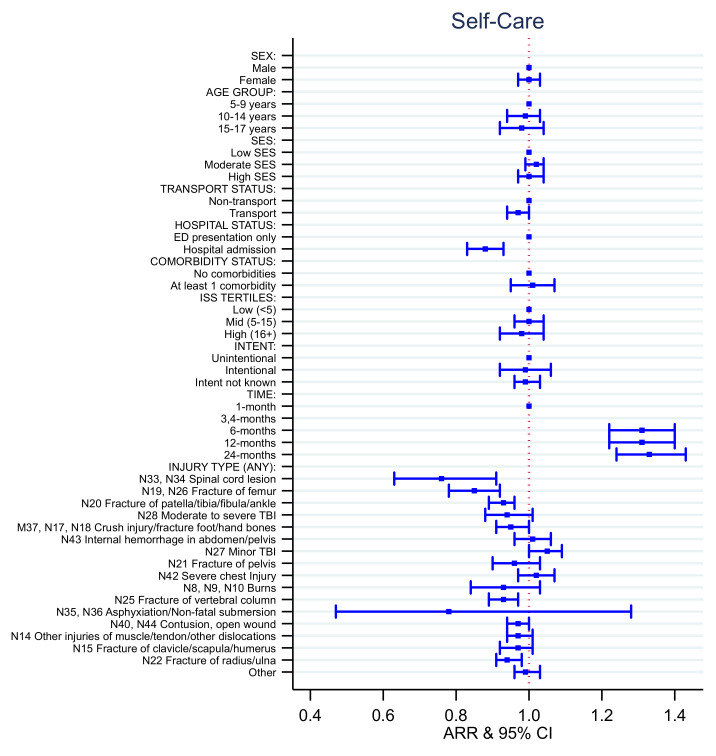
EQ-5D self-care adjusted relative risk (ARR).

**Figure 6:**
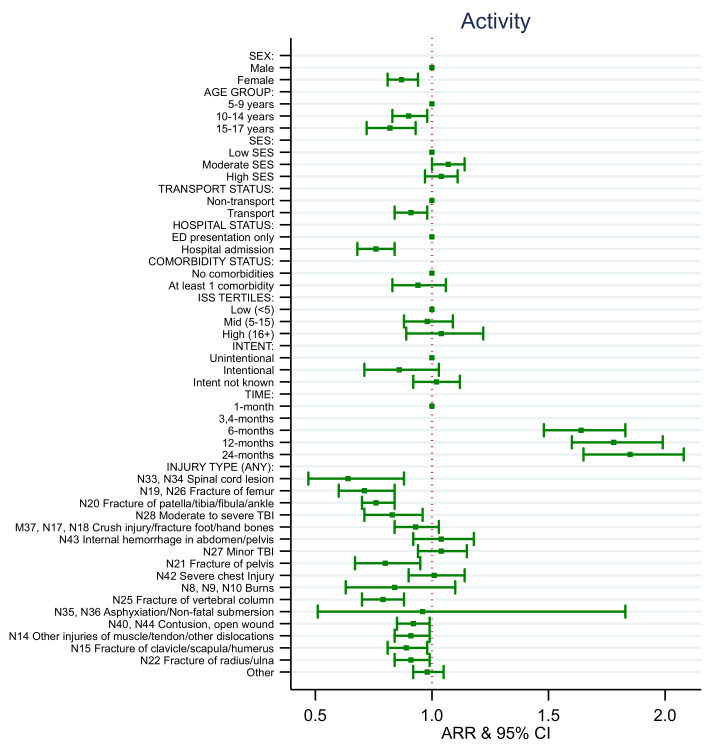
EQ-5D activity adjusted relative risk (ARR).

**Figure 7:**
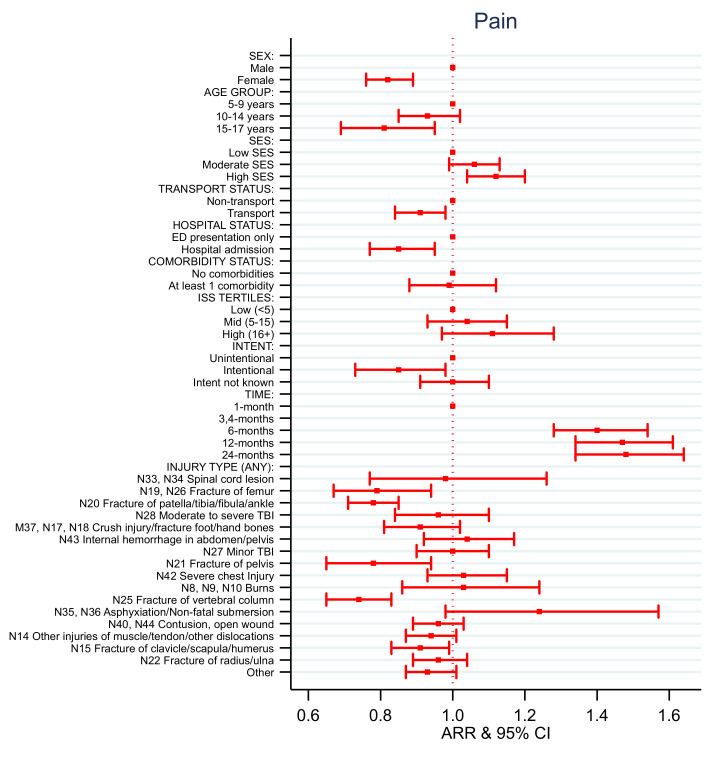
EQ-5D pain adjusted relative risk (ARR).

**Figure 8:**
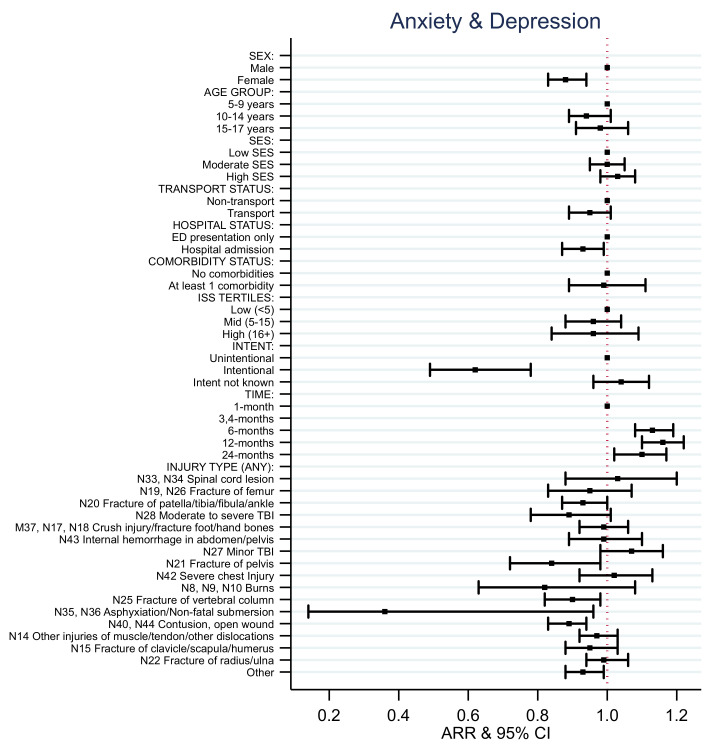
EQ-5D anxiety and depression adjusted relative risk (ARR).

Consistent with the results for the EQ-5D utility score, the adjusted relative risk (ARR) of reporting no problems after injury with mobility, activity, pain, and anxiety and depression for females was lower than for males over time (Supplementary Table S3, Figure 4, Figure 5, Figure 6, Figure 7 and Figure 8). Children aged 10–14 years of age and aged 15–17 years of age had decreased rates of ARR or reporting no problems with activity post injury over time compared to those aged 5–9 years of age (Supplementary Table S3, Figure 6). Children aged 15–17 years of age had a 19% decreased rate of ARR or reporting no problems with pain post injury over time compared to those aged 5–9 years of age (Supplementary Table S3, Figure 7). Children from moderate SES had a 7% increased ARR of reporting no problems with activity over time compared to children from low SES and children from high SES areas had a 12% increased ARR of reporting no problems with pain over time post injury compared to children with low SES (Supplementary Table S3, Figure 6 and Figure 7).

The ARR of reporting no problems varied depending on the injury sustained and health states (Supplementary Table S3, Figure 4, Figure 5, Figure 6, Figure 7 and Figure 8). The ARR of reporting no problems after injury was lower for the transport group compared to the non-transport group across all health states, except for anxiety and depression. Children admitted to hospital had a decreased ARR over time of reporting no problems across all health states compared to children with ED presentations only. Children involved in intentional injuries had a 15% and 38% decreased ARR over time post-injury of reporting no problems with pain, and with anxiety and depression, respectively, compared to children who had unintentional injuries.

The ARR of reporting no problems across the health states for children who had sustained a fracture of the femur, patella/tibia/fibula/ankle, pelvis, clavicle/scapula/humerus and/or vertebral column differed. Children who sustained a fracture of femur had decreased rates of ARR and reporting no problems over time with mobility (29%), self-care (15%), activity (29%), and pain (21%). Children who sustained a fracture of patella/tibia/fibula/ankle had a decreased ARR of reporting no problems over time with mobility (23%), self-care (7%), activity (24%), pain (12%), and anxiety and depression (7%). Children who sustained a fracture of the pelvis had a decreased ARR of reporting no problems over time with mobility (16%), activity (20%), pain (12%), and anxiety and depression (16%). Children who sustained a fracture of the vertebral column had a decreased ARR of reporting no problems over time with self-care (7%), activity (21%), pain (26%), and anxiety and depression (10%). Children who sustained a fracture of the clavicle/scapula/humerus had a decreased ARR of reporting no problems over time with activity (11%) and pain (9%). Children who sustained a fracture of the radius or ulna had a decreased ARR of reporting no problems over time with self-care (6%) and activity (9%).

Children who had sustained a spinal cord injury had a decreased ARR of reporting no problems over time with mobility (39%), self-care (24%), and activity (36%) compared to children who had not sustained this type of injury. Children who had sustained a moderate to severe traumatic brain injury had a 17% decreased ARR of reporting no problems over time with activity compared to children who had not sustained this type of injury. Children who had sustained a non-fatal submersion injury resulting in asphyxiation had a 64% decreased ARR of reporting no problems over time with anxiety and depression compared to children who had not sustained this type of injury.

Children who sustained a contusion or open wound had a decreased ARR of reporting no problems over time with mobility (5%), self-care (3%), activity (8%), and anxiety and depression (11%) compared to children who had not sustained these types of injury. Children who had sustained other injuries of muscle and tendon and other dislocations had a decreased ARR of reporting no problems over time with mobility (5%) and activity (9%) compared to children who had not sustained this type of injury.

### 4.5. HRQoL Trajectories

**Figure 9:**
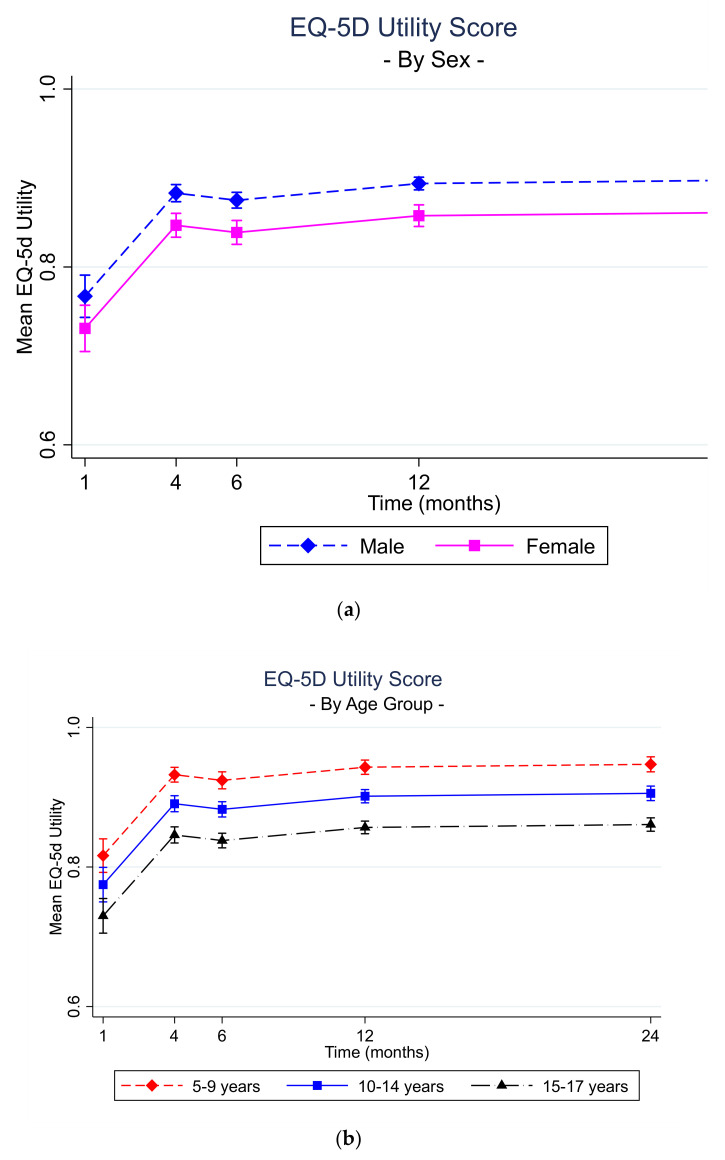

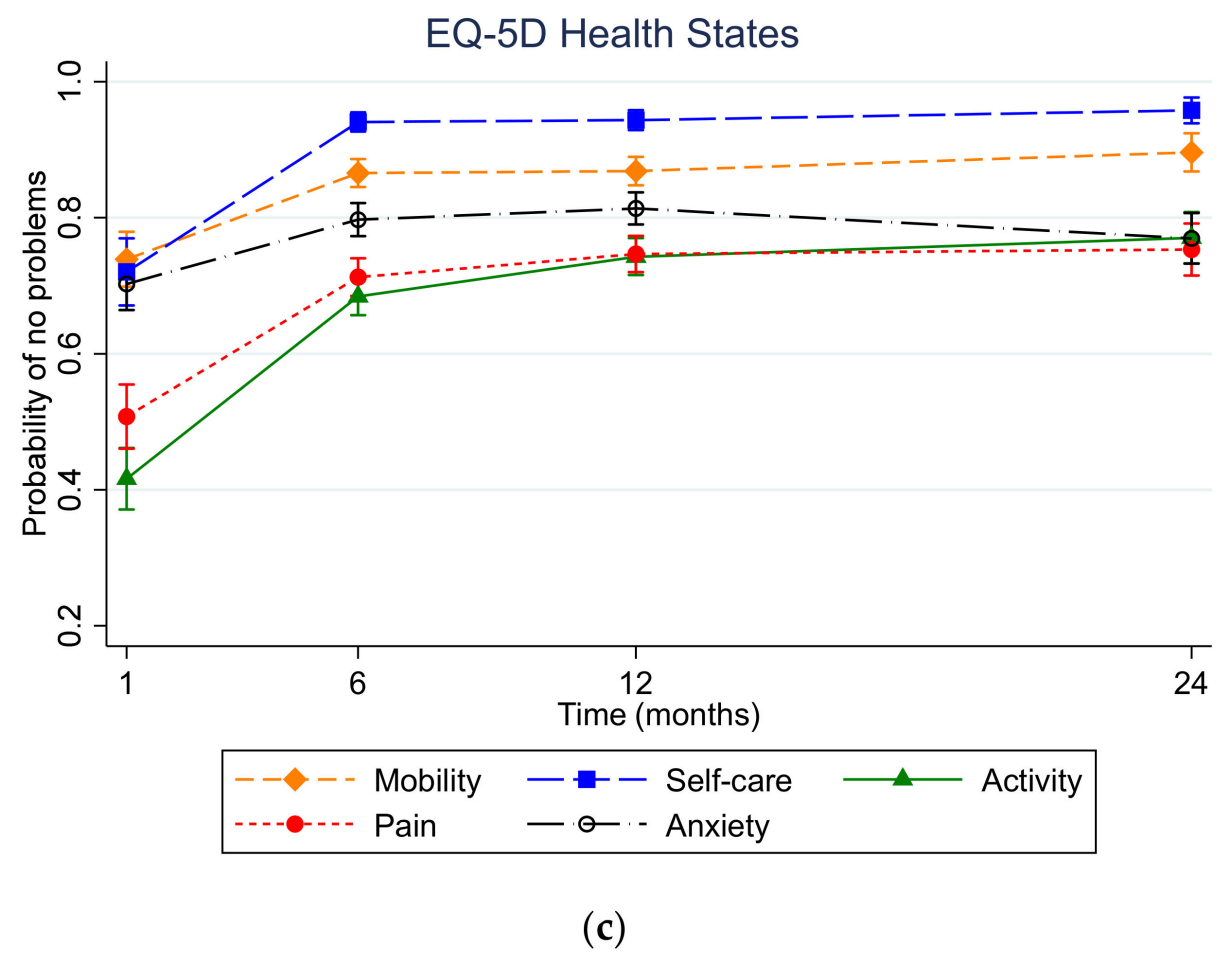
Predicted Margins: (**a**) EQ-5D utility score across time by sex; (**b**) EQ-5D utility score across time by age group; (**c**) EQ-5D individual health states across time.

The HRQoL trajectories for females from 4 months to 24 months post injury were lower than for males (Figure 9). In general, the older the child, the worse the trajectory from 4 months to 24 months post injury. None of the health state trajectories reached full health (i.e., probability of no problems, or 1) by 24 months. However, there were differences across the health state trajectories with the probability of no problems with self-care rising quickly from 1 month to 4 months and plateauing from 6 months to 24 months. The probability of reporting no problems with mobility increased from 1 month to 4 months and again from 12 months to 24 months. The probability of reporting no problems with activity rose from 1 month to 24-but the trajectory is lowest across all health states at 1 and 4 months, lower than three of the four health states at 12 months and remains lower than mobility and self-care at 24 months. Whilst the probability of no problems with pain increased from 1 month to 12 months, it fell from 12 months to 24 months and remained lower than mobility and self-care at 24 months. The probability of no problems with anxiety and depression increased from 1 month to 12 months but decreased by 24 months to remain lower than mobility and self-care at 24 months.

## 5. Discussion

This study found distinct patterns of HRQoL trajectories for children and adolescents following injury across the five health states of the EQ-5D. This indicates the need for intervention strategies that take account of varying profiles in post-injury recovery among children by demographic and injury categories. The recovery of children and adolescents following injury differs according to their sex, age, SES, and the nature of the injury sustained. The observed lack of continuity across health state trajectories following injury has highlighted the complexity of recovery in this population.

Of concern, this study found that none of the HRQoL health state trajectories for children and adolescents following injury reached full health (i.e., probability of no problems = 1) by 24 months. Based on the health state, HSDC and HRQoL trajectory analysis, 12 months post-injury seems to be a key time point. The proportion of children with a complete health state response pattern with no problems (i.e., 11111) was highest at 12 months but then dropped at 24 months. The HSDC graph indicated the health states were less evenly spread at 12 months and the rise at 24 months, which included problems with pain, may be a reason for this change from 12 to 24 months. Specific demographic and injury-related attributes were found to have a decreased relative risk of no problems with pain over time post injury. Some of the reasons for these research findings may be that the injury-related deficits may become more noticeable as the physical and psychosocial demands on the child increase as the child gets older. There is also the possibility that functioning really plateaus from 12 to 24 months, but parental expectations and ratings change during this time period. By 24 months post-injury, parents may be more concerned about the ongoing and long-lasting limitations resulting from their child’s injury. Earlier on in the child’s recovery, parents may be more optimistic when rating their child’s functionality with the hope that their child will recover. As parents witness their child becoming less socially active, their concerns about entrenched difficulties may translate into more pessimistic reports regarding their child’s limitations at 24 months.

Children from moderate and high SES areas had a higher HRQoL on average post-injury over time compared to children from low SES areas. This finding is consistent with systematic reviews of HRQoL and functional outcomes in children after injury [28,29]. There was a 12% higher relative risk over time of reporting no pain problems for children and adolescents from high SES areas compared to children and adolescents from low SES areas. The additional family stressors, such as financial, social, employment, and health issues [30,31] may impact a child’s recovery from injury. SES has negative effects on the wellbeing and development of children and adolescents as has been shown repeatedly in all studies that have examined this. Children from low SES backgrounds are less likely to have access to healthcare [32]. Issues such as access to a regular doctor [33], or services such as physiotherapy, and the financial costs of pain medications could hinder a child’s pain management.

The variability in the patterns of recovery in HRQoL health states for children and adolescents sustaining fractures is of interest. For example, children who sustained a fracture of the patella, tibia, fibula, or ankle had a reduced relative risk of having no problems over time across all five health states, and children who sustained a fracture of the femur had a reduced relative risk of having no problems over time across all health states, except anxiety and depression. A fracture of the lower limb in children and adolescents limits their mobility and activity as weight-bearing activity could be limited. In some instances, surgery may be required to treat growth plate damage (e.g., in the tibia) and this type of damage cannot be detected until a year or longer after injury, which may further limit a child’s activity. In contrast, children who sustained a fracture of the pelvis had a reduced relative risk of having no problems over time with mobility, activity, pain, and/or anxiety and depression. This type of facture is often caused by motor vehicle injuries, which are often high impact traumatic events. There is a strong association between operative treatment and the severity of the pelvic injury, with displaced pelvic fractures in children having the potential to lead to pelvic asymmetry and thereby worse outcomes [34].

This study found the probability of reporting no problems with self-care rose quickly from 1 month to 4 months and then plateaued to 24 months. Children who sustained a spinal cord injury had a reduced risk of having no problems with self-care over time. Children sustaining spinal cord injuries frequently endure significant impairments in physical functioning, often requiring extensive nursing (e.g., bowel and bladder management, pressure ulcer prevention, and medical complications such as autonomic dysreflexia) [35] which reduces their own self-care. Compounding this are the physical changes children undergo as they progress into adulthood.

The probability of no problems with anxiety and depression increased from 1 month to 12 months but decreased by 24 months. Females had a reduced risk over time of reporting no problems with anxiety and depression compared to males. This finding is not surprising as the prevalence of depression is higher in females than males, and commonly increases in females after puberty [36] To review this, an interaction between age and sex was included in the models (Supplementary Table S4) and a significant interaction was found for 15–17-year-old females compared to males. Children who had sustained an intentional injury, asphyxiation, or non-fatal submersion injury also had a reduced risk over time of reporting no problems with anxiety and depression. This is consistent with research reporting raised prevalence of depression among patients who experienced an event such as interpersonal violence. [37]

This study had a number of strengths: pooling of injury-specific and primary data of patient-centered outcomes at a number of time points post injury and a robust sample size that permitted disaggregated analyses covering the most common injury categories in the GBD 2013. The limitations of this study mainly reflect the complexities of pooling data from multiple international data sets: differing follow-up points and unbalanced sample sizes across these time points; differing inclusion criteria, which resulted in differing proportions of cases with certain injuries (e.g., TBI); multiple injuries across the datasets; different calendar years; differing types of healthcare systems and levels of health insurance; and the exclusion of the CHAI cohort from the health states analysis. However, our models accounted for a variety of injuries and adjusted for data sources to ensure estimates were independent of these inherent differences in time and setting. The mapping of the CHAI PedsQL to the EQ-5D utility score did allow us to measure HRQoL overall. Even though the HRQoL measures may have some heterogeneity across the five cohorts and over time, the cohort was included as a control in the models and robust standard errors were adjusted for any potential heteroscedasticity. As the potential covariate of race/ethnicity was not consistently collected in each cohort, our models were unable to explore the association between this social determinant and the HRQoL outcomes. This study reports factors influencing the HRQoL outcomes of children and adolescents following injury in high income countries. It is acknowledged that treatment services used after injury (e.g., mental health or services) will likely impact on a child’s functional outcomes. As this information was not available for all cohorts, further research is warranted to understand the impact of service utilization on child injury outcomes. More research attention to these influences is particularly warranted in low- and middle-income countries where the nature of injuries can vary and economic resources, health, and rehabilitation systems are more constrained.

## 6. Conclusions

This study identified demographic and injury related predictors and trajectories for HRQoL outcomes over time for children and adolescents following injury. The HRQoL recovery trajectories for this study population do not reach full health by 24 months, plateauing from 6 to 24 months. Different intervention strategies are required to deal with recovery from injury in children and adolescents and to address different recovery profiles in demographic and injury sub-groups and different trajectories in the health states of mobility, self-care, activity, pain, and anxiety and depression. This study will enable better recognition and understanding of the individual and societal impacts of injury in children and adolescents over time and provides a basis for better estimates of the burden of injury. The different patterns of health state recovery trajectories identified in this study have the potential to guide prioritization of prevention efforts and inform health and social service planning to reduce post injury HRQoL deficits experienced by children and adolescents.

## Figures and Tables

**Table 1:** Specific details of VIBES-Junior cohorts.

Study & Setting	Month/Year & Participants	Inclusion Criteria & Injury Diagnosis Coding	Post-Injury Follow-Up Time Point EQ-5D Measures & Mode of Interview
VSTR Australia	03/2009 to 03/2017 *n* = 824, 5–16 years	In hospital death, ISS > 12, ICU admission or urgent surgery, met burns criteria 20–29% full/partial thickness. ICD-10-AM	EQ-5D total score and items at 6, 12 and 24 months. Telephone
VOTOR Australia	03/2009 to 03/2017 *n* = 502, 16–17 years	16+ years of age, orthopedic injury admission >24 h or death ICD-10-AM	EQ-5D total score and items at 6, 12 and 24 months. Telephone
CHAI United States of America	03/2007 to 09/2008 *n* = 635, 5–17 years	Presentation to ED or hospital admission for either a TBI or an upper extremity injury ICD-9 mapped to ICD-10	PedsQL scores mapped to EQ-5D total score at 3, 12, and 24 months. Online, telephone and postal
UKBOI United Kingdom	09/2005 to 04/2007 *n* = 174, 5–17 years	Presentation to ED or hospital admission. ICD-10	EQ-5D total score and items at 1, 6 and 12 months. Postal
BCCH-LIO Canada	02/2011 to 12/2013 *n* = 199, 5–16 years	Presentation to ED or hospital admission. ICD-10	EQ-5D total score and items at 1, 4, and 12 months. Postal and online

**Table 2:** VIBES-Junior pooled cohort characteristics included in analysis and lost to follow-up for EQ-5D (*n* = 2734).

	Lost to Follow Up (*n* = 400)	Included (*n* = 2334)	Total (*n* = 2734)	*p*-Value ^†^
**Sex**				0.162
Male	279 (69.8%)	1710 (73.3%)	1989 (72.8%)	
Female	121 (30.2%)	624 (26.7%)	745 (27.2%)	
**Age Group**				<0.001
5–9 years	190 (47.5%)	371 (15.9%)	561 (20.5%)	
10–14 years	82 (20.5%)	728 (31.2%)	810 (29.6%)	
15–17 years	128 (32.0%)	1235 (52.9%)	1363 (49.9%)	
**Age (years) (mean, SD)**	10.8 (4.4)	13.6 (3.5)	13.1 (3.8)	
**Socio Economic Status (SES) Tertile**				0.608
Low SES	155 (39.9%)	842 (37.8%)	997 (38.1%)	
Moderate SES	151 (38.9%)	926 (41.5%)	1077 (41.2%)	
High SES	82 (21.1%)	461 (20.7%)	543 (20.7%)	
**Cohort**				<0.001
VSTR	270 (67.5%)	824 (35.3%)	1094 (40.0%)	
VOTOR	49 (12.2%)	502 (21.5%)	551 (20.2%)	
CHAI	12 (3.0%)	635 (27.2%)	647 (23.7%)	
UKBOI	7 (1.8%)	174 (7.5%)	181 (6.6%)	
BCCH-LIO	62 (15.5%)	199 (8.5%)	261 (9.5%)	
**Transport Status**				0.136
Non-transport	226 (58.9%)	1438 (63.0%)	1664 (62.4%)	
Transport	158 (41.1%)	845 (37.0%)	1003 (37.6%)	
**Hospital Status**				<0.001
ED only	50 (12.5%)	641 (27.5%)	691 (25.3%)	
Hospital admission	350 (87.5%)	1691 (72.5%)	2041 (74.7%)	
**Comorbidity status**				<0.001
No comorbidities	340 (85.0%)	2140 (91.7%)	2480 (90.7%)	
At least 1 comorbidity	60 (15.0%)	194 (8.3%)	254 (9.3%)	
**ISS Tertile**				<0.001
Low (<5)	92 (23.4%)	1035 (45.4%)	1127 (42.1%)	
Mid (5–15)	119 (30.2%)	620 (27.2%)	739 (27.6%)	
High (16+)	183 (46.4%)	625 (27.4%)	808 (30.2%)	
**Intent**				0.032
Unintentional	332 (83.0%)	1855 (79.5%)	2187 (80.0%)	
Intentional	24 (6.0%)	110 (4.7%)	134 (4.9%)	
Intent not known	44 (11.0%)	369 (15.8%)	413 (15.1%)	
**Injury Type (any) ^^^**				
N33, N34 spinal cord lesion	8 (2.0%)	45 (1.9%)	53 (1.9%)	1.000
N19, N26 fracture of femur	30 (7.5%)	121 (5.2%)	151 (5.5%)	0.079
N20 fracture of patella, tibia, fibula, or ankle	30 (7.5%)	274 (11.7%)	304 (11.1%)	0.016
N28 moderate to severe traumatic brain injury	122 (30.5%)	424 (18.2%)	546 (20.0%)	<0.001
N37, N17, N18 crush injury, fracture foot/hand bones	16 (4.0%)	150 (6.4%)	166 (6.1%)	0.078
N43 internal hemorrhage in abdomen or pelvis	85 (21.2%)	323 (13.8%)	408 (14.9%)	<0.001
N27 minor traumatic brain injury	64 (16.0%)	614 (26.3%)	678 (24.8%)	<0.001
N21 fracture of pelvis	34 (8.5%)	159 (6.8%)	193 (7.1%)	0.266
N42 severe chest Injury	60 (15.0%)	272 (11.7%)	332 (12.1%)	0.070
N8, N9, N10 burns (including lower airways)	20 (5.0%)	35 (1.5%)	55 (2.0%)	<0.001
N25 fracture of vertebral column	45 (11.2%)	268 (11.5%)	313 (11.4%)	0.960
N35, N36 asphyxiation, Non-fatal submersion	<10	<10	12 (0.4%)	0.542
N40, N44 contusion, open wound	117 (29.2%)	537 (23.0%)	654 (23.9%)	0.008
N14 other injuries of muscle & tendon and other dislocations	63 (15.8%)	297 (12.7%)	360 (13.2%)	0.116
N15 fracture of clavicle, scapula, or humerus	36 (9.0%)	255 (10.9%)	291 (10.6%)	0.286
N22 fracture of radius or ulna	35 (8.8%)	325 (13.9%)	360 (13.2%)	0.006
Other	228 (57.0%)	1003 (43.0%)	1231 (45.0%)	<0.001

Note: Overall proportion lost to follow up = 14.6%. SES = socio economic status, ISS = injury severity score, ED = emergency department. Missing data for the included sample: SES *n* = 104, transport status *n* = 51, hospital status *n* < 5, SES, ISS *n* = 54. ^^^ Injury groupings based on GBD 2013 classifications with reference = does not have injury type. The order follows GBD 2013 categories. ^†^ *p*-values from Pearson’s chi-square for categorical variables and ANOVA for continuous variables.

## Data Availability

The data presented in this study are available on request from the relevant custodian. The data are not publicly available due to ethical and privacy issues.

## Supplementary Materials

The following are available online at https://www.mdpi.com/article/10.3390/ijerph181910156/s1, Table S1: EQ-5D-3L Health State Questions and Response Options, Table S2: Mean and Standard Deviation (SD) of EQ-5D Utility Score and Frequency and Percentages of EQ-5D Health States Over Time, Table S3: EQ-5D Utility Score and Five Health States Model Results, Table S4: EQ-5D Utility Score and Five Health States Model Results Including Age × Sex Interaction.
