# Supplementary material for: Health-Related Quality of Life (HRQoL) Outcomes Following Injury in Childhood and Adolescence Using EuroQol (EQ-5D) Responses with Pooled Longitudinal Data
Source: Int J Environ Res Public Health. 2021 Sep 27;18(19):10156. doi: 10.3390/ijerph181910156 (PMC8507627; doi:10.3390/ijerph181910156)
Supplement: Supplementary file 1 [file ijerph-18-10156-s001.zip › ijerph-1339012-supplementary.pdf]

International Journal of
Environmental Research and Public Health

SUPPLEMENTARY MATERIAL

Supplementary Table S1. EQ-5D-3L Health State Questions and Response Options.

**EQ-5D-3L**
**Mobility (walking about)**
I have no problems walking about
I have some problems walking about
I am confined to bed
**Self-care**
I have no problems with self-care
I have some problems washing or dressing myself
I am unable to wash or dress myself
**Usual activities (e.g. work, study, housework, family or leisure activities)**
I have no problems with performing my usual activities
I have some problems with performing my usual activities
I am unable to perform my usual activities
**Pain/discomfort**
I have no pain or discomfort
I have moderate pain or discomfort
I have extreme pain or discomfort
**Anxiety/depression**
I am not anxious or depressed
I am moderately anxious or depressed
I am extremely anxious or depressed

Supplementary Table S2. Mean and Standard Deviation (SD) of EQ-5D Utility Score and Frequency and Percentages of EQ-5D Health States Over Time.

Measure	Baseline	1 month	4 months	6 months	12 months	24 months
**EQ-5D Utility Score**	**N=1071**	**n=344**	**n=784**	**n=1309**	**n=2000**	**n=1362**
Mean (SD)	0.947 (0.091)	0.818 (0.222)	0.896 (0.134)	0.858 (0.187)	0.887 (0.159)	0.884 (0.151)
**EQ-5D - Mobility**	**n=427**	**n=351**		**n=1127**	**n=1083**	**n=533**
Problems	9 (2.1%)	68 (19.4%)		142 (12.6%)	125 (11.5%)	66 (12.4%)
No Problems	418 (97.9%)	283 (80.6%)		985 (87.4%)	958 (88.5%)	467 (87.6%)
**EQ-5D – Self-Care**	**n=429**	**n =353**		**n=1116**	**n=1070**	**n=533**
Problems	8 (1.9%)	85 (24.1%)		64 (5.7%)	54 (5.0%)	29 (5.4%)
No Problems	421 (98.1%)	268 (75.9%)		1052 (94.3%)	1016 (95.0%)	504 (94.6%)
**EQ-5D - Activity**	**n=430**	**n=352**		**n=1107**	**n=1061**	**n=533**
Problems	17 (4.0%)	164 (46.6%)		323 (29.2%)	243 (22.9%)	144 (27.0%)
No Problems	413 (96.0%)	188 (53.4%)		784 (70.8%)	818 (77.1%)	389 (73.0%)
**EQ-5D - Pain**	**n=428**	**n=359**		**n=1095**	**n=1051**	**n=526**
Problems	25 (5.8%)	139 (38.7%)		297 (27.1%)	245 (23.3%)	144 (27.4%)
No Problems	403 (94.2%)	220 (61.3%)		798 (72.9%)	806 (76.7%)	382 (72.6%)
**EQ-5D – Anxiety and Depression**	**n=427**	**n=357**		**n=1128**	**n=1080**	**n=529**
Problems	19 (4.5%)	63 (17.6%)		206 (18.3%)	179 (16.6%)	142 (26.8%)
No Problems	408 (95.5%)	294 (82.4%)		922 (81.7%)	901 (83.4%)	387 (73.2%)

Supplementary Table S3. EQ-5D Utility Score and Five Health States Model Results.

	EQ-5D Utility Score UCoeff (CI)	EQ-5D Utility Score ACoef. (CI)	Mobility URR (CI)	Mobility ARR (CI)	Self-Care URR (CI)	Self-Care ARR (CI)	Activity URR (CI)	Activity ARR (CI)	Pain URR (CI)	Pain ARR (CI)	Anxiety & Depression URR (CI)	Anxiety & Depression ARR (CI)
**Sex**												
Male	Ref											
Female	**-0.03 (-0.05,-0.02)**	**-0.04 (-0.05,-0.02)**	0.97 (0.93,1.01)	0.95~ (0.91,1.00)	1.01 (0.98,1.04)	1.00 (0.97,1.03)	0.93~ (0.86,1.00)	**0.87 (0.81,0.94)**	**0.86 (0.80,0.93)**	**0.82 (0.76,0.89)**	**0.90 (0.85,0.96)**	**0.88 (0.83,0.94)**
**Age Group**												
5-9 years	Ref											
10-14 years	**-0.04 (-0.06,-0.03)**	**-0.03 (-0.05,-0.02)**	0.97 (0.92,1.02)	0.97 (0.93,1.02)	0.98 (0.94,1.03)	0.99 (0.94,1.03)	**0.92 (0.85,0.99)**	**0.90 (0.83,0.98)**	0.94 (0.87,1.02)	0.93 (0.85,1.02)	0.97 (0.92,1.01)	0.94 (0.89,1.01)
15-17 years	**-0.09 (-0.1,-0.08)**	**-0.06 (-0.08,-0.05)**	**0.87 (0.83,0.90)**	0.95 (0.86,1.04)	**0.93 (0.91,0.96)**	0.98 (0.92,1.04)	**0.71 (0.67,0.76)**	**0.82 (0.72,0.93)**	**0.76 (0.71,0.81)**	**0.81 (0.69,0.95)**	**0.82 (0.78,0.86)**	0.98 (0.91,1.06)
**Socio Economic Status (SES)**												
Low SES	Ref											
Moderate SES	**0.03 (0.01,0.04)**	0.02~ (0.00,0.03)	1.01 (0.97,1.06)	1.01 (0.97,1.05)	1.02 (0.99,1.04)	1.02 (0.99,1.04)	**1.09 (1.02,1.17)**	**1.07 (1.00,1.14)**	1.06 (0.99,1.13)	1.06 (0.99,1.13)	1.02 (0.97,1.08)	1.00 (0.95,1.05)
High SES	**0.05 (0.03,0.07)**	**0.03 (0.01,0.04)**	**1.05 (1.00,1.10)**	1.03 (0.99,1.08)	1.02 (0.98,1.05)	1.00 (0.97,1.04)	**1.10 (1.02,1.18)**	1.04 (0.97,1.11)	**1.16 (1.08,1.24)**	**1.12 (1.04,1.20)**	**1.09 (1.03,1.15)**	1.03 (0.98,1.08)
**Transport Status**												
Non-transport	Ref											
Transport	**-0.07 (-0.08,-0.05)**	**-0.02 (-0.04,-0.01)**	**0.86 (0.82,0.90)**	**0.92 (0.88,0.97)**	**0.93 (0.91,0.96)**	0.97~ (0.94,1.00)	**0.76 (0.71,0.81)**	**0.91 (0.84,0.98)**	**0.78 (0.73,0.83)**	**0.91 (0.84,0.98)**	**0.84 (0.79,0.88)**	0.95 (0.89,1.01)
**Hospital Status**												
ED presentation only	Ref											
Hospital admission	**-0.06 (-0.07,-0.05)**	0.00 (-0.02,0.02)	**0.85 (0.82,0.87)**	**0.93 (0.88,0.99)**	**0.89 (0.87,0.91)**	**0.88 (0.83,0.93)**	**0.68 (0.65,0.71)**	**0.76 (0.68,0.84)**	**0.76 (0.72,0.80)**	**0.85 (0.77,0.95)**	**0.81 (0.78,0.84)**	**0.93 (0.87,0.99)**
**Comorbidity Status**												
No comorbidities	Ref											
At least 1 comorbidity	**-0.09 (-0.11,-0.06)**	**-0.04 (-0.06,-0.01)**	**0.85 (0.77,0.94)**	0.99 (0.90,1.08)	**0.93 (0.87,0.99)**	1.01 (0.95,1.07)	**0.78 (0.68,0.88)**	0.94 (0.83,1.06)	**0.88 (0.78,0.99)**	0.99 (0.88,1.12)	**0.78 (0.69,0.89)**	0.99 (0.89,1.11)
**ISS Tertiles**												
Low (<5)	Ref											
Mid (5-15)	**-0.08 (-0.09,-0.06)**	-0.02~ (-0.04,0.00)	**0.89 (0.85,0.93)**	0.99 (0.93,1.06)	**0.94 (0.91,0.96)**	1.00 (0.96,1.04)	**0.74 (0.69,0.80)**	0.98 (0.88,1.09)	**0.79 (0.74,0.85)**	1.04 (0.93,1.15)	**0.78 (0.74,0.83)**	0.96 (0.88,1.04)
High (16+)	**-0.07 (-0.09,-0.06)**	-0.01 (-0.03,0.02)	**0.85 (0.8,0.90)**	0.95 (0.87,1.05)	**0.90 (0.87,0.94)**	0.98 (0.92,1.04)	**0.72 (0.66,0.78)**	1.04 (0.89,1.22)	**0.85 (0.79,0.91)**	1.11 (0.97,1.28)	**0.75 (0.7,0.81)**	0.96 (0.84,1.09)

**Intent**												
Unintentional	Ref											
Intentional	**-0.09**	**-0.08**	0.97	0.94	1.00	0.99	0.86~	0.86	0.90	**0.85**	**0.57**	**0.62**
	**(-0.13,-0.06)**	**(-0.11,-0.04)**	(0.9,1.06)	(0.86,1.03)	(0.95,1.05)	(0.92,1.06)	(0.73,1.00)	(0.71,1.03)	(0.79,1.03)	**(0.73,0.98)**	**(0.46,0.71)**	**(0.49,0.78)**
Intent not known	**0.03**	0.02	1.02	1.00	**1.03**	0.99	**1.08**	1.02	**1.06**	1.00	**1.10**	1.04
	**(0.01,0.04)**	(-0.01,0.04)	(0.98,1.06)	(0.94,1.07)	**(1.01,1.05)**	(0.96,1.03)	**(1.02,1.14)**	(0.92,1.12)	**(1.00,1.12)**	(0.91,1.10)	**(1.06,1.15)**	(0.96,1.12)
**Time**												
1-month	Ref											
3,4-months	**0.10**	**0.12**	NA	NA	NA	NA	NA	NA	NA	NA	NA	NA
	**(0.08,0.12)**	**(0.09,0.14)**										
6-months	**0.08**	**0.11**	**1.08**	**1.17**	**1.24**	**1.31**	**1.33**	**1.64**	**1.19**	**1.40**	0.99	**1.13**
	**(0.06,0.10)**	**(0.08,0.13)**	**(1.03,1.14)**	**(1.11,1.23)**	**(1.17,1.32)**	**(1.22,1.40)**	**(1.2,1.46)**	**(1.48,1.83)**	**(1.09,1.29)**	**(1.28,1.54)**	(0.94,1.05)	**(1.08,1.19)**
12-months	**0.11**	**0.13**	**1.10**	**1.18**	**1.25**	**1.31**	**1.44**	**1.78**	**1.25**	**1.47**	1.01	**1.16**
	**(0.09,0.13)**	**(0.1,0.15)**	**(1.04,1.16)**	**(1.11,1.24)**	**(1.18,1.33)**	**(1.22,1.40)**	**(1.31,1.60)**	**(1.6,1.99)**	**(1.15,1.36)**	**(1.34,1.61)**	(0.96,1.07)	**(1.1,1.22)**
24-months	**0.11**	**0.13**	**1.09**	**1.21**	**1.25**	**1.33**	**1.37**	**1.85**	**1.19**	**1.48**	**0.89**	**1.10**
	**(0.09,0.13)**	**(0.11,0.15)**	**(1.02,1.15)**	**(1.14,1.29)**	**(1.17,1.33)**	**(1.24,1.43)**	**(1.22,1.53)**	**(1.65,2.08)**	**(1.07,1.31)**	**(1.34,1.64)**	**(0.83,0.95)**	**(1.02,1.17)**
**Injury Type (any)^**												
N33, N34 Spinal cord lesion	**-0.16**	**-0.11**	**0.62**	**0.61**	**0.74**	**0.76**	**0.56**	**0.64**	0.85	0.98	0.96	1.03
	**(-0.23,-0.09)**	**(-0.17,-0.04)**	**(0.45,0.85)**	**(0.45,0.83)**	**(0.60,0.90)**	**(0.63,0.91)**	**(0.40,0.78)**	**(0.47,0.88)**	(0.68,1.06)	(0.77,1.26)	(0.84,1.11)	(0.88,1.20)
N19, N26 Fracture of femur	**-0.11**	**-0.07**	**0.68**	**0.71**	**0.83**	**0.85**	**0.64**	**0.71**	**0.76**	**0.79**	**0.87**	0.95
	**(-0.14,-0.08)**	**(-0.10,-0.04)**	**(0.59,0.79)**	**(0.62,0.82)**	**(0.77,0.90)**	**(0.78,0.92)**	**(0.54,0.76)**	**(0.60,0.84)**	**(0.64,0.89)**	**(0.67,0.94)**	**(0.77,0.98)**	(0.83,1.07)
N20 Fracture of patella, tibia, fibula, or ankle	**-0.05**	**-0.06**	**0.79**	**0.77**	**0.96**	**0.93**	**0.83**	**0.76**	**0.82**	**0.78**	0.98	0.93~
	**(-0.08,-0.03)**	**(-0.08,-0.04)**	**(0.74,0.85)**	**(0.72,0.83)**	**(0.93,0.99)**	**(0.89,0.96)**	**(0.76,0.91)**	**(0.70,0.84)**	**(0.75,0.89)**	**(0.71,0.85)**	(0.92,1.04)	(0.87,1.00)
N28 Moderate to severe traumatic brain injury	**-0.04**	-0.02~	0.94	0.95	0.95~	0.94	**0.75**	**0.83**	**0.89**	0.96	**0.77**	0.89
	**(-0.05,-0.02)**	(-0.04,0.00)	(0.88,1.01)	(0.87,1.03)	(0.9,1.00)	(0.88,1.01)	**(0.66,0.85)**	**(0.71,0.96)**	**(0.81,0.98)**	(0.84,1.10)	**(0.70,0.86)**	(0.78,1.01)
N37, N17, N18 Crush injury, fracture foot/hand bones	-0.03	**-0.01**	0.92~	0.93	0.96	0.95~	0.93	0.93	0.89~	0.91	0.98	0.99
	(-0.06,0.00)	**(-0.03,0.02)**	(0.85,1.00)	(0.87,1.01)	(0.91,1.01)	(0.91,1.00)	(0.83,1.04)	(0.84,1.03)	(0.79,1.00)	(0.81,1.02)	(0.91,1.07)	(0.92,1.06)
N43 Internal hemorrhage in abdomen or pelvis	-0.02~	0.01	**0.92**	0.98	0.97	1.01	0.90~	1.04	0.96	1.04	**0.90**	0.99
	(-0.04,0.00)	(-0.02,0.03)	**(0.85,0.99)**	(0.9,1.06)	(0.93,1.01)	(0.96,1.06)	(0.82,1.00)	(0.92,1.18)	(0.87,1.05)	(0.92,1.17)	**(0.82,0.98)**	(0.89,1.10)
N27 Minor TBI	-0.01	0.00	0.99	1.05	1.00	**1.05**	**0.88**	1.04	**0.91**	1.00	**0.90**	1.07
	(-0.02,0.01)	(-0.01,0.02)	(0.94,1.04)	(0.99,1.12)	(0.97,1.04)	**(1.00,1.09)**	**(0.80,0.97)**	(0.94,1.15)	**(0.83,0.99)**	(0.90,1.10)	**(0.84,0.97)**	(0.98,1.16)

N21 Fracture of pelvis	**-0.11**	**-0.05**	**0.77**	**0.84**	**0.92**	0.96	**0.63**	**0.80**	**0.65**	**0.78**	**0.70**	**0.84**
	**(-0.15,-0.08)**	**(-0.08,-0.02)**	**(0.68,0.88)**	**(0.74,0.94)**	**(0.86,0.99)**	(0.90,1.03)	**(0.52,0.77)**	**(0.67,0.95)**	**(0.54,0.79)**	**(0.65,0.94)**	**(0.59,0.82)**	**(0.72,0.98)**
N42 Severe chest Injury	**-0.06**	0.01	**0.90**	1.03	**0.94**	1.02	**0.78**	1.01	**0.84**	1.03	**0.82**	1.02
	**(-0.08,-0.04)**	(-0.01,0.03)	**(0.84,0.97)**	(0.96,1.10)	**(0.90,0.98)**	(0.97,1.07)	**(0.69,0.87)**	(0.90,1.14)	**(0.76,0.93)**	(0.93,1.15)	**(0.75,0.90)**	(0.92,1.13)
N8, N9, N10 Burns (including lower airways)	-0.05	-0.06	0.97	0.96	0.97	0.93	0.92	0.84	1.06	1.03	0.87	0.82
	(-0.12,0.01)	(-0.13,0.01)	(0.84,1.12)	(0.85,1.09)	(0.88,1.07)	(0.84,1.03)	(0.71,1.19)	(0.63,1.10)	(0.88,1.27)	(0.86,1.24)	(0.69,1.10)	(0.63,1.08)
N25 Fracture of vertebral column	**-0.10**	**-0.05**	**0.88**	0.95	**0.90**	**0.93**	**0.71**	**0.79**	**0.71**	**0.74**	**0.84**	**0.90**
	**(-0.12,-0.07)**	**(-0.08,-0.03)**	**(0.83,0.95)**	(0.90,1.02)	**(0.86,0.94)**	**(0.89,0.97)**	**(0.64,0.80)**	**(0.70,0.88)**	**(0.64,0.79)**	**(0.65,0.83)**	**(0.78,0.91)**	**(0.82,0.98)**
N35, N36 Asphyxiation, Non-fatal submersion	**-0.04**	**-0.12**	0.97	0.83	0.98	0.78	0.92	0.96	0.94	1.24	0.97	**0.36**
	**(-0.25,-0.1)**	**(-0.21,-0.03)**	(0.44,1.22)	(0.57,1.22)	(0.54,1.21)	(0.47,1.28)	(0.63,1.45)	(0.51,1.83)	(1.01,1.40)	(0.98,1.57)	(0.18,1.07)	**(0.14,0.96)**
N40, N44 Contusion, open wound	**-0.06**	-0.02~	**0.89**	**0.95**	**0.95**	0.97~	**0.80**	**0.92**	**0.84**	0.96	**0.79**	**0.89**
	**(-0.07,-0.04)**	(-0.03,0.00)	**(0.85,0.94)**	**(0.91,0.99)**	**(0.92,0.98)**	(0.94,1.00)	**(0.74,0.86)**	**(0.85,0.99)**	**(0.78,0.90)**	(0.89,1.03)	**(0.74,0.85)**	**(0.83,0.94)**
N14 Other injuries of muscle & tendon and other dislocations	**-0.03**	-0.02~	**0.92**	0.95~	0.97~	0.97	**0.91**	**0.91**	**0.92**	0.94	0.97	0.97
	**(-0.05,-0.01)**	(-0.04,0.00)	**(0.87,0.98)**	(0.90,1.00)	(0.94,1.00)	(0.94,1.01)	**(0.84,0.99)**	**(0.84,0.99)**	**(0.85,0.99)**	(0.87,1.01)	(0.92,1.03)	(0.92,1.03)
N15 Fracture of clavicle, scapula, or humerus	-0.02~	-0.02~	1.01	0.99	0.98	0.97	0.92	**0.89**	0.93	**0.91**	0.95	0.95
	(-0.05,0.00)	(-0.04,0.00)	(0.95,1.06)	(0.94,1.04)	(0.94,1.02)	(0.92,1.01)	(0.83,1.02)	**(0.81,0.98)**	(0.85,1.02)	**(0.83,0.99)**	(0.87,1.02)	(0.88,1.03)
N22 Fracture of radius or ulna	0.02	0.00	1.09	0.98	0.98	**0.94**	1.07	**0.91**	1.11	0.96	1.09	0.99
	(0.01,0.04)	(-0.02,0.02)	(1.05,1.13)	(0.94,1.02)	(0.95,1.01)	**(0.91,0.98)**	(0.99,1.15)	**(0.84,0.99)**	(1.03,1.18)	(0.89,1.04)	(1.04,1.15)	(0.94,1.06)
Other	**-0.05**	-0.01~	**0.94**	0.99	**0.97**	0.99	**0.84**	0.98	**0.85**	0.93	**0.81**	**0.93**
	**(-0.06,-0.04)**	(-0.03,0.00)	**(0.90,0.98)**	(0.95,1.04)	**(0.95,0.99)**	(0.96,1.03)	**(0.79,0.89)**	(0.92,1.05)	**(0.8,0.90)**	(0.87,1.01)	**(0.77,0.85)**	**(0.88,0.99)**
Number of observations		5,341		2,776		2,759		2,747		2,723		2,776
Number of groups		2,135		1,131		1,133		1,131		1,134		1,134

Note: Ref=Reference group, UCoef=Unadjusted Coefficient, ACoef= Adjusted Coefficient, URR=Unadjusted Relative Risk of No problems, ARR=Adjusted Relative Risk of No problems, CI=95% Confidence Interval, ED=Emergency Department, ISS=Injury Severity Score. ^ = Reference group for each injury group is absence of the injury group. ~ This measure was considered important as showed a positive 95% CI. Intraclass correlation (ICC) was 0.60. Significant figures in Bold.

Supplementary Table S4. EQ-5D Utility Score and Five Health States Model Results Including Age x Sex Interaction

	EQ-5D Utility Score ACoef. (CI)	Mobility ARR (CI)	Self-Care ARR (CI)	Activity ARR (CI)	Pain ARR (CI)	Anxiety & Depression ARR (CI)
**Sex**						
Male	Ref					
Female	-0.01	0.96	0.96	0.96	0.91	0.99
	(-0.03, 0.01)	(0.91, 1.02)	(0.90, 1.02)	(0.85, 1.08)	(0.80, 1.03)	(0.91, 1.08)
**Age Group**						
5-9 years	Ref					
10-14 years	-0.03	0.97	0.96	0.92	0.94	0.96
	(-0.05, -0.01)	(0.93, 1.02)	(0.91, 1.02)	(0.83, 1.02)	(0.84, 1.04)	(0.88, 1.05)
15-17 years	-0.05	0.96	0.96	0.87	0.87	1.06
	(-0.07, -0.03)	(0.87, 1.06)	(0.89, 1.03)	(0.75, 1.01)	(0.73, 1.03)	(0.97, 1.16)
**Sex x Age Group Interaction**						
Female x 5-9 years	Ref					
Female x 10-14 years	0.00	1.01	1.05	1.04	1.07	1.06
	(-0.03, 0.03)	(0.92, 1.11)	(0.97, 1.15)	(0.89, 1.22)	(0.89, 1.28)	(0.96, 1.18)
Female x 15-17 years	**-0.05**	0.98	1.04	0.87	0.85	**0.82**
	**(-0.08, -0.02)**	(0.90, 1.07)	(0.97, 1.13)	(0.74, 1.01)	(0.72, 1.01)	**(0.73, 0.93)**
**Socio Economic Status (SES)**						
Low SES	Ref					
Moderate SES	0.01	1.01	1.01	**1.07**	1.06	1.00
	(0.00, 0.03)	(0.97, 1.05)	(0.99, 1.04)	**(1.01, 1.14)**	(0.99, 1.13)	(0.95, 1.05)
High SES	**0.03**	1.03	1.00	1.04	**1.12**	1.03
	**(0.01, 0.04)**	(0.99, 1.08)	(0.97, 1.04)	(0.97, 1.11)	**(1.05, 1.20)**	(0.98, 1.08)
**Transport Status**						
Non-transport	Ref					
Transport	**-0.02**	**0.92**	0.97	**0.91**	**0.91**	0.95
	**(-0.04, -0.01)**	**(0.88, 0.97)**	(0.94, 1.00)	**(0.84, 0.98)**	**(0.84, 0.98)**	(0.89, 1.01)
**Hospital Status**						
ED presentation only	Ref					
Hospital admission	0.00	0.94	**0.88**	**0.77**	**0.88**	0.95
	(-0.02, 0.02)	(0.88, 1.00)	**(0.83, 0.93)**	**(0.69, 0.87)**	**(0.79, 0.98)**	(0.89, 1.01)
**Comorbidity Status**						
No comorbidities	Ref					
At least 1 comorbidity	**-0.04**	0.99	1.01	0.94	0.99	0.99
	**(-0.06, -0.01)**	(0.90, 1.08)	(0.95, 1.07)	(0.83, 1.06)	(0.88, 1.12)	(0.89, 1.11)
**ISS Tertiles**						
Low (<5)	Ref					
Mid (5-15)	-0.02	0.99	1.00	0.98	1.04	0.97
	(-0.04, 0.00)	(0.93, 1.07)	(0.96, 1.04)	(0.88, 1.09)	(0.94, 1.15)	(0.89, 1.05)
High (16+)	**-0.01**	0.95	0.98	1.04	1.11	0.96
	**(-0.03, 0.02)**	(0.87, 1.05)	(0.92, 1.04)	(0.89, 1.21)	(0.96, 1.28)	(0.84, 1.09)
**Intent**						
Unintentional	Ref					
Intentional	**-0.08**	0.94	0.99	0.85	**0.85**	**0.62**
	**(-0.11, -0.04)**	(0.86, 1.03)	(0.92, 1.06)	(0.71, 1.02)	**(0.73, 0.98)**	**(0.49, 0.78)**
Intent not known	0.02	1.00	0.99	1.02	1.00	1.04
	(-0.01, 0.04)	(0.94, 1.07)	(0.96, 1.03)	(0.92, 1.12)	(0.91, 1.10)	(0.96, 1.12)
**Time**						
1-month	Ref					
3,4-months	**0.12**					

	**(0.09, 0.14)**					
6-months	**0.11**	**1.17**	**1.31**	**1.64**	**1.40**	**1.13**
	**(0.08, 0.13)**	**(1.11, 1.23)**	**(1.22, 1.40)**	**(1.48, 1.82)**	**(1.28, 1.53)**	**(1.08, 1.19)**
12-months	**0.13**	**1.18**	**1.31**	**1.78**	**1.47**	**1.16**
	**(0.10, 0.15)**	**(1.11, 1.24)**	**(1.22, 1.40)**	**(1.60, 1.98)**	**(1.34, 1.61)**	**(1.10, 1.22)**
24-months	**0.13**	**1.21**	**1.33**	**1.85**	**1.48**	**1.09**
	**(0.11, 0.15)**	**(1.14, 1.29)**	**(1.24, 1.43)**	**(1.64, 2.07)**	**(1.33, 1.64)**	**(1.02, 1.17)**
**Injury Type (any)^**						
N33, N34 Spinal cord lesion	**-0.11**	**0.61**	**0.76**	**0.64**	0.98	1.02
	**(-0.18, -0.04)**	**(0.45, 0.83)**	**(0.63, 0.91)**	**(0.47, 0.87)**	(0.76, 1.26)	(0.87, 1.19)
N19, N26 Fracture of femur	**-0.07**	**0.71**	**0.85**	**0.71**	**0.79**	0.95
	**(-0.10, -0.04)**	**(0.62, 0.82)**	**(0.78, 0.92)**	**(0.60, 0.84)**	**(0.67, 0.94)**	(0.84, 1.07)
N20 Fracture of patella, tibia, fibula, or ankle	**-0.06**	**0.77**	**0.93**	**0.76**	**0.78**	0.93
	**(-0.08, -0.04)**	**(0.72, 0.83)**	**(0.89, 0.96)**	**(0.70, 0.84)**	**(0.71, 0.86)**	(0.87, 1.00)
N28 Moderate to severe traumatic brain injury	-0.02	0.95	0.94	**0.83**	0.96	0.89
	(-0.04, 0.00)	(0.87, 1.03)	(0.88, 1.01)	**(0.71, 0.96)**	(0.85, 1.10)	(0.79, 1.01)
N37, N17, N18 Crush injury, fracture foot/hand bones	-0.01	0.93	0.95	0.93	0.91	0.99
	(-0.03, 0.02)	(0.87, 1.01)	(0.91, 1.00)	(0.84, 1.03)	(0.81, 1.02)	(0.92, 1.06)
N43 Internal hemorrhage in abdomen or pelvis	0.01	0.98	1.01	1.04	1.04	0.99
	(-0.02, 0.03)	(0.90, 1.06)	(0.96, 1.06)	(0.92, 1.17)	(0.92, 1.16)	(0.89, 1.10)
N27 Minor TBI	0.00	1.05	1.05	1.04	1.00	1.07
	(-0.01, 0.02)	(0.99, 1.12)	(1.00, 1.09)	(0.94, 1.15)	(0.91, 1.10)	(0.99, 1.16)
N21 Fracture of pelvis	**-0.05**	**0.84**	0.96	**0.80**	**0.79**	**0.85**
	**(-0.08, -0.02)**	**(0.74, 0.95)**	(0.90, 1.03)	**(0.67, 0.96)**	**(0.66, 0.95)**	**(0.73, 0.99)**
N42 Severe chest Injury	0.01	1.03	1.02	1.01	1.03	1.02
	(-0.01, 0.03)	(0.96, 1.10)	(0.97, 1.07)	(0.90, 1.14)	(0.93, 1.15)	(0.92, 1.13)
N8, N9, N10 Burns (including lower airways)	-0.06	0.96	0.93	0.84	1.04	0.82
	(-0.13, 0.01)	(0.85, 1.09)	(0.84, 1.03)	(0.63, 1.11)	(0.86, 1.25)	(0.63, 1.08)
N25 Fracture of vertebral column	**-0.05**	0.95	**0.93**	**0.79**	**0.74**	**0.90**
	**(-0.07, -0.03)**	(0.90, 1.02)	**(0.89, 0.97)**	**(0.71, 0.89)**	**(0.65, 0.84)**	**(0.83, 0.99)**
N35, N36 Asphyxiation, Non-fatal submersion	**-0.11**	0.84	0.78	0.98	1.27	**0.37**
	**(-0.20, -0.03)**	(0.57, 1.23)	(0.47, 1.28)	(0.51, 1.88)	(0.99, 1.63)	**(0.14, 0.95)**
N40, N44 Contusion, open wound	**-0.02**	**0.95**	0.97	0.92	0.96	**0.89**
	**(-0.03, -0.00)**	**(0.91, 0.99)**	(0.94, 1.00)	(0.85, 1.00)	(0.89, 1.03)	**(0.83, 0.95)**
N14 Other injuries of muscle & tendon and other dislocations	**-0.02**	0.95	0.97	**0.91**	0.94	0.97
	**(-0.04, 0.00)**	(0.90, 1.00)	(0.94, 1.01)	**(0.84, 0.99)**	(0.87, 1.01)	(0.92, 1.03)
N15 Fracture of clavicle, scapula, or humerus	-0.02	0.99	0.97	**0.89**	0.91	0.95
	(-0.04, 0.00)	(0.94, 1.04)	(0.92, 1.01)	**(0.81, 0.98)**	(0.83, 1.00)	(0.88, 1.03)
N22 Fracture of radius or ulna	-0.00	0.98	**0.94**	**0.91**	0.97	1.00
	(-0.02, 0.02)	(0.94, 1.02)	**(0.90, 0.97)**	**(0.84, 0.99)**	(0.89, 1.05)	(0.94, 1.06)
Other	**-0.01**	0.99	0.99	0.98	0.93	**0.93**
	**(-0.03, -0.00)**	(0.95, 1.04)	(0.96, 1.03)	(0.91, 1.05)	(0.87, 1.00)	**(0.87, 0.98)**

Number of observations	5,341	2,776	2,759	2,747	2,723	2,776
Number of groups	2,135	1,131	1,133	1,131	1,134	1,134

Note: Ref=Reference group, ACoef= Adjusted Coefficient, ARR=Adjusted Relative Risk of No problems, CI=95% Confidence Interval, ED=Emergency Department, ISS=Injury Severity Score. ^ = Reference group for each injury group is absence of the injury group. Significant figures in Bold. Significant interaction shaded gray.
